# Suburban Road Networks to Explore COVID-19 Vulnerability and Severity

**DOI:** 10.3390/ijerph19042039

**Published:** 2022-02-11

**Authors:** Shahadat Uddin, Arif Khan, Haohui Lu, Fangyu Zhou, Shakir Karim

**Affiliations:** School of Project Management, Faculty of Engineering, The University of Sydney, Forest Lodge, NSW 2037, Australia; arif.khan@sydney.edu.au (A.K.); haohui.lu@sydney.edu.au (H.L.); fangyu.zhou@sydney.edu.au (F.Z.); shakir.karim@sydney.edu.au (S.K.)

**Keywords:** suburban road network, centrality, COVID-19 Delta variant, vulnerability and severity

## Abstract

The Delta variant of COVID-19 has been found to be extremely difficult to contain worldwide. The complex dynamics of human mobility and the variable intensity of local outbreaks make measuring the factors of COVID-19 transmission a challenge. The inter-suburb road connection details provide a reliable proxy of the moving options for people between suburbs for a given region. By using such data from Greater Sydney, Australia, this study explored the impact of suburban road networks on two COVID-19-related outcomes measures. The first measure is COVID-19 vulnerability, which gives a low score to a more vulnerable suburb. A suburb is more vulnerable if it has the first COVID-19 case earlier and vice versa. The second measure is COVID-19 severity, which is proportionate to the number of COVID-19-positive cases for a suburb. To analyze the suburban road network, we considered four centrality measures (degree, closeness, betweenness and eigenvector) and core–periphery structure. We found that the degree centrality measure of the suburban road network was a strong and statistically significant predictor for both COVID-19 vulnerability and severity. Closeness centrality and eigenvector centrality were also statistically significant predictors for COVID-19 vulnerability and severity, respectively. The findings of this study could provide practical insights to stakeholders and policymakers to develop timely strategies and policies to prevent and contain any highly infectious pandemics, including the Delta variant of COVID-19.

## 1. Introduction

SARS-CoV-2 (severe acute respiratory syndrome coronavirus 2) is a novel coronavirus that was first reported in the Hubei Province of the People’s Republic of China in late December 2019. It has since spread to most countries, including Australia. The WHO Emergency Committee declared a global health emergency on 30 January 2020, citing rising cases in Chinese and international locales. Coronavirus is a broad family of viruses that can cause illnesses ranging from the common cold to more severe conditions, such as severe acute respiratory syndrome [1]. The case-detection rate fluctuates daily and, for Australia, it can be monitored in real-time on the Australian NSW Health and COVID Live website and other platforms, such as Data.NSW [2]. Coronavirus has four genera: alpha-, beta-, gamma-, and delta-coronaviruses. While alpha- and beta-coronaviruses are found in mammals, especially bats, gamma- and delta-coronaviruses are found in pigs and birds [3]. The beta-coronavirus genus can infect humans and cause serious sickness and even death, whereas alpha-coronavirus induces asymptomatic or minimally symptomatic infections [2,3]. The current COVID-19 pandemic is caused by the beta-coronavirus.

COVID-19 symptoms can develop within two to fourteen days of exposure. A patient can also be infectious before any symptoms appear—known as the pre-symptomatic spread. Symptoms range in severity and affect people differently. For most cases, the illness is mild to moderate and requires no hospitalization. However, COVID-19 poses a greater risk of serious illness in the elderly that increases with age [4]. People with pre-existing medical conditions or chronic diseases may be at a higher risk of developing a severe illness [4].

COVID-19 disproportionately impacts vulnerable populations, such as the elderly, people with disabilities, people in prison, Aboriginal and Torres Strait Islander communities, people with chronic conditions, and people from culturally and linguistically diverse backgrounds [5,6]. In many countries, access to primary and secondary healthcare is not government-subsidized, and the cost and availability of healthcare are often out of reach for the most vulnerable [7,8]. Furthermore, the elderly and disabled people rely on public transport for essential services, such as grocery shopping and healthcare during pandemics [9,10]. People from various sociodemographic and low socioeconomic backgrounds and numerous racial and ethnic minorities are unlikely to have the financial resources to make self-distancing and self-isolation a viable option [11,12]. Furthermore, if not treated carefully, self-isolating aspirations worsen mental health difficulties among the most vulnerable communities [13,14]. As a result, many governments are facing difficulty taking initiatives to lower health inequalities by addressing social determinants of health.

Since the onset of the pandemic, a tremendous global scholarly effort has been put into modeling the pandemic and predicting transmission. Using sociodemographic and COVID-19-specific themes, Tiwari et al. [15] proposed a random-forest machine-learning-based vulnerability model. The COVID-19 impact assessment technique incorporates homogeneity and trend analysis, which aid in determining the severity of the pandemic and training the vulnerability model. Another study [16] applied a machine-learning-based improved model to analyze and predict the growth of the epidemic and the possible threat of COVID-19 in nations around the world. Prout et al. [17] have employed a random-forest machine-learning technique to find the strongest predictors of distress. They also developed regression trees to identify individuals at higher risk for anxiety, depression, and post-traumatic stress.

Most related work has explored historical COVID-19 infection data with both classical mechanistic models and machine-learning models. Similar to effective forecasting models for other epidemics, Hernandez-Matamoros [18] assessed the autoregressive integrated moving average (ARIMA) model for 145 countries in six regions, and this turned out to be also effective for COVID-19. Swaraj [19] proposed an ARIMA-based model that could capture the linear and non-linear components of the data by integrating an autoregressive neural network (NAR). Machine-learning and deep-learning models have also shown outstanding ability to forecast from time-series data, especially Long Short-Term Memory (LSTM), due to their capability to unveil dependencies over a long distance in time. Hssayeni [20] employed the LSTM model on a county level. Kim’s two-level LSTM-based model [21], Hi-COVIDNet, gave better performance by combining country-level and continent-level encoders.

Intuitively, mobility data are closely related to the spread of epidemics. COVID-19 is no exception. Badr et al. [22] presented daily mobility data derived from aggregated cell-phone data and fitted a statistical model for each county. Nevertheless, many network-based models have been introduced to analyze COVID-19 spread across different levels of areas because these models are deemed to capture mobility data characteristics better. Chang et al. [23] proposed a metapopulational susceptible–exposed–infectious–removed (SEIR) model that integrates dynamic mobility networks consisting of movements from CBGs (census block groups) to POIs (point of interests). The social-network-based model introduced by Block et al. [24] demonstrates that reduced contact between people improves the effectiveness of social-distancing strategies. Many other mobility simulations have shown a strong correlation between human movement and the spread of COVID-19, even without proper models to analyze the prediction [25].

While many studies explored the relation between mobility and COVID-19 propagation, they primarily focused on larger geographic areas, e.g., cities, states, etc. [26,27], and predicting case counts. However, this approach often ignores the more granular movements of people across the suburbs through local roads and the chances of introducing the virus to adjacent suburbs. This study aims to fill in this gap. We looked at both suburb-level road connections and sociodemographic factors to explore how these affect the chance of getting the first COVID-19 case (vulnerability) when adjacent suburbs are infected, as well as the severity of cases after having the first case in the Australian context.

## 2. Materials and Methods

### 2.1. Data Source

This study considered data from 19 local government areas (LGA) of Greater Sydney of New South Wales, Australia. These LGAs were severely affected by the Delta variant of COVID-19, and the first locally acquired case was identified on 16 June 2021 [28]. There are 137 postal areas in these 19 LGAs. Supplementary Table S1 briefly summarizes the sociodemographic information of these postal areas. The data in this table are extracted from the Australian Bureau of Statistics [29]. We used Google maps to calculate road connectivities between two suburbs. All research methods were performed in accordance with relevant guidelines and regulations.

### 2.2. Suburban Road Network: Construction and Analysis

**Figure 1:**
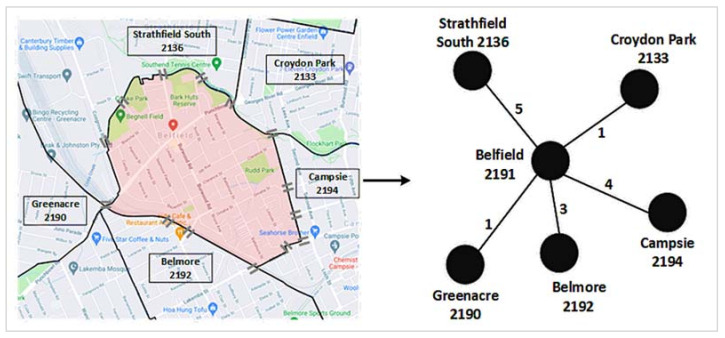
A suburban road network construction: Left: Google map of Belfield (shaded by light red color) and its road connections with five other neighboring suburbs. The ‘=’ sign represents a road connection between two suburbs. Right: The corresponding road network. The edge weight between Belfield and Campsie is 4, since four roads connect these two suburbs. We repeated these two steps for each suburb considered in this study to have the final suburban road network, as presented in in the results section.

A network is a collection of nodes and edges, where nodes represent different entities and edges indicate connections between two nodes [30]. A suburban road network is a network of suburbs that are connected through one or more roads. We consider postcodes as nodes to construct the suburban road network, i.e., a postcode represents a suburb. An edge between two suburbs demonstrates that at least a road connects them directly without going through any intermediate suburb. We used the number of roads connecting two suburbs as their edge weight. Figure 1 illustrates the construction process of a suburban road network, using data from Google maps.

We used centrality measures and core–periphery structure to analyze the suburban road network. The four centrality measures that we considered are degree, closeness, betweenness, and eigenvector. The degree centrality of a node in a network indicates its direct connectivity with other network nodes [30]. A node with a higher number of connections to other network nodes will have a higher degree centrality and vice versa. The closeness centrality represents the reachability of a node from other nodes of the network [30]. A node that is easily reachable by other network nodes will have a higher closeness centrality value and vice versa. The betweenness centrality is based on the shortest paths, and it gives a node a higher score if that node falls in the shortest paths of any other pair of nodes more often [30]. The eigenvector centrality is also known as the ‘prestige score’. A high eigenvector centrality for a node means that it is connected to many other nodes that have high scores [30]. Further details of each of these four centrality measures are presented in Supplementary Figure S1.

We then used the core–periphery structure to explore the coreness of each node of the suburban road network. Core–periphery structure analysis of a network assigns a coreness score to each node of that network [30]. A node tightly connected with other network nodes has a higher coreness value and vice versa. Using an abstract network dataset, Supplementary Figure S2 illustrates the analysis of a core–periphery structure.

### 2.3. COVID-19 Vulnerability and Severity

This study used two COVID-19-related measures for each suburb: vulnerability and severity. The vulnerability score of a suburb is the date difference between the first date when a locally acquired COVID-19 Delta variant case was found in that suburb and the ‘date zero’. The ‘date zero’ is the date (i.e., 16 June 2021) when the first locally acquired COVID-19 variant was found within the 19 LGAs considered in this study. A suburb will have a higher vulnerability score if it has its first locally acquired COVID-19 Delta case lately. A low score for this measure indicates that the underlying suburb is more vulnerable to COVID-19. The severity measure of a suburb is the total number of locally acquired COVID-19 Delta variant cases per thousand population as of the last date of our data collection period. A higher value for this measure indicates that COVID-19 has badly infected the underlying postal area or suburb.

## 3. Results

**Figure 2:**
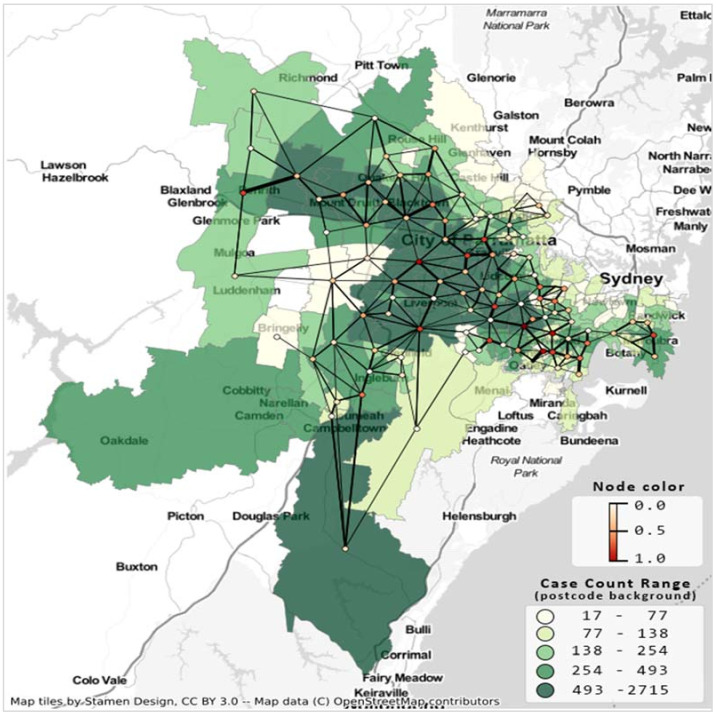
Suburb road network and COVID-19 case count Greater Sydney LGAs for the second wave of COVID-19, case count up to 10 October 2021. Postcode area’s shade indicates case count, network’s node color indicates degree centrality, and edge thickness is proportionate to the number of shared roads between the corresponding postal regions.

Figure 2 presents the resultant undirected suburban road network considering 137 postal areas from 19 LGAs. The minimum and maximum edge weights are 1 and 18, respectively. This undirected network has a density of 0.04; that is, only 4% of edges (405) are present in the network out of 4658 possible edges.

### 3.1. Correlation Results

**Figure 3:**
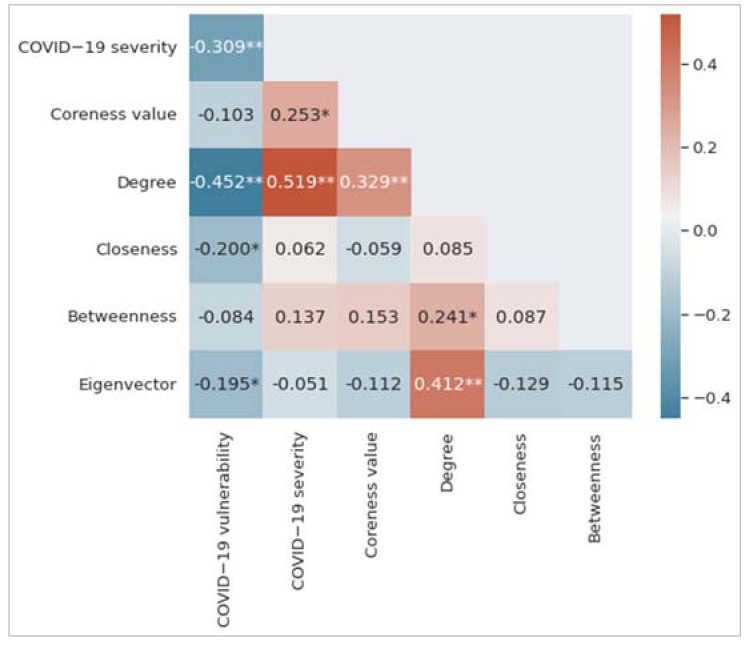
Correlation matrix among the variables considered in this study. Significance levels of 0.01 and 0.05 have been represented by two asterisks (**) and one asterisk (*), respectively.

Figure 3 shows the correlation matrix, which is the summary of the correlations between variables. Each of the colored cells shows a correlation coefficient value between two variables. The cell color represents the strength of the correlation. As can be seen, we find that degree centrality has the highest correlation coefficient value with both COVID-19 vulnerability and COVID-19 severity measures. It has a negative correlation with COVID-19 vulnerability (r = −0.452, *p* < 0.01) and a positive correlation with COVID-19 severity (r = 0.519, *p* < 0.01). The COVID-19 vulnerability measure negatively correlates with closeness centrality (r = −0.200, *p* < 0.05) and eigenvector centrality (r = −0.195, *p* < 0.01). The coreness measure positively correlates with the COVID-19 severity measure (r = 0.253, *p* < 0.05). A low value of the COVID-19 vulnerability measure indicates that the underlying postal area or suburb is more vulnerable to COVID-19; however, the COVID-19 severity measure is proportionate to the number of individuals identified by COVID-19 in a postal area.

### 3.2. Regression Results

Table 1 shows the results from the multiple linear regression (MLR) model for the COVID-19 vulnerability measure. The corresponding coefficient of determination (R^2^ value) is 23.30%. Assessing only the *p*-values suggests that degree centrality and closeness centrality are statistically significant at *p* < 0.05. The t-statistics magnitude can be used to assess the relative relevance of the independent variables. In this case, degree centrality is the most significant independent variable, followed by closeness centrality. Although eigenvector centrality has a significant negative correlation (from Figure 3) with COVID-19 vulnerability, it does not reach the required statistical significance level in the MLR model.

The MLR model of Table 2 reveals that degree centrality and eigenvector centrality are statistically significant predictors for the COVID-19 severity measure. The perceived coefficient of determination (R^2^ value) is 35.80%. The eigenvector centrality did not correlate with the COVID-19 severity measure, as per Figure 3. However, this variable is found as a statistically significant predictor for the COVID-19 severity measure because the degree centrality variable acted as a suppressor for the eigenvector centrality in the corresponding MLR model. A suppressor is a variable that can influence the relationship between a predictor and an outcome measure when added to a regression model [31]. As per the t-statistics, the most significant predictor is the degree centrality measure, followed by the eigenvector centrality measure.

**Figure 4:**
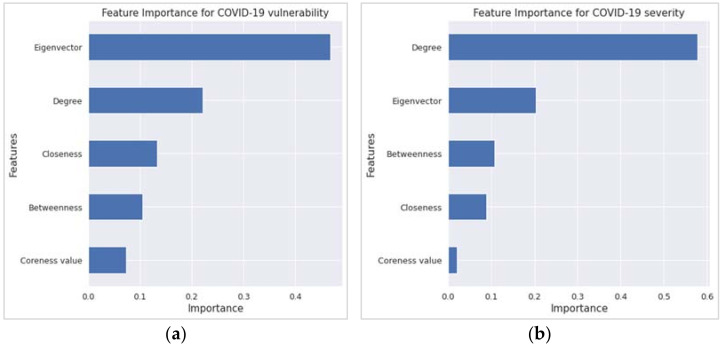
Feature importance results from the random forest regression for (**a**) COVID-19 vulnerability and (**b**) COVID-19 severity.

Concerning the variable or feature importance, the random forest regression (RFR) findings echoed the corresponding findings from the MLR. The two most important features in Figure 4a,b, which have an importance score of ≥0.20, are the first two variables according to the t-statistics order in Table 1 and Table 2. However, the R^2^ value from the RFR is higher than that of the MLR, as revealed in Table 3.

## 4. Discussion and Conclusions

Network measures of the suburban road network have been found to be an important predictor of COVID-19 vulnerability and severity. Degree centrality is an important predictor for both COVID-19 vulnerability and severity. Closeness centrality and eigenvector centrality can predict COVID-19 vulnerability and severity, respectively. Apparently, this is the first study to explore COVID-19 vulnerability and severity by using suburban road networks.

Although the eigenvector was not a significant predictor in the MLR model, it has become the most important variable, followed by degree and closeness for vulnerability in the random forest regression. This is because feature importance is not a measure of effect size in random-forest algorithms. It is a measurement of a variable’s contribution to out-of-bag prediction performance. A variable can be relevant in a random forest because of how it interacts with other factors and separates the data independently. Nothing prevents a non-significant variable (i.e., eigenvector) in a regression model from having high importance in the random forest [31]. Indeed, even within the regression framework, nothing prevents excluding a variable with a small effect size from having a large effect on predictive accuracy, especially if that variable has strong confounding effects on other predictors. Inclusion of those confounding effects leads to better predictions [31].

A postal area with comparatively more connections to other postal areas will have a higher degree centrality value in the corresponding suburban road network. People living in that postal area will therefore have more options to move to the neighboring postal areas. Similarly, there is a higher chance that more people from those neighboring postal areas will visit this postal area. Such opportunities will eventually put the people of this postal area in more danger in terms of being infected by COVID-19 earlier (if not infected yet) and a quicker COVID-19 spread among the community (once it is identified). This is what is reflected in this study’s findings. For example, the degree centrality measure negatively correlates with the COVID-19 vulnerability measure since a low value of the latter indicates that the underlying suburb is more vulnerable to COVID-19. Closeness centrality is a distance-based measure of a network. It puts a suburb or postal area of the suburban road network in a position that is easily reachable by others. This means that the people of that suburb are comfortably reachable by the people of other suburbs. In the same way, people of that suburb can reach others easily. Such a reachable status makes people more vulnerable to being infected sooner by any super-infectious diseases, including COVID-19.

Relevant stakeholders and policymakers could consider this study’s findings in developing strategies to prevent the spread and reduce the severity of any pandemics, including COVID-19. During Sydney’s second wave of the COVID-19 Delta variant, the State Government first imposed a local lockdown within a radius of 10 km and then within a 5-km boundary on different dates. For example, for Sydney and its neighboring LGAs, the 10-km restriction was imposed on 9 July 2021 [32]. According to this study, such lockdowns might not be fully effective to stop the highly contagious virus from spreading to neighboring suburbs. The reason is that many of those LGAs under lockdown have neighboring suburbs or postal areas within a 10 km or 5 km radius. These restrictions, therefore, would not stop people from moving in and out across infected suburbs, thus bringing the virus to newer suburbs, as we have seen in our study case of the Delta outbreak in Sydney, Australia. Fortunately, the outbreak did not go out of control, as the social distancing, mask mandates, frequent testing, and lockdown measures were followed well. However, for more transmissible variants (such as Omicron) or for any future cases, the finding of this study gives evidence of suburb-wide lockdown as an effective containment measure.

Although this study considered only one study site from a country, the proposed study design, based on the suburban road network, is original and can be followed to explore the vulnerability and severity of COVID-19 (or other pandemics) in any other study areas from the same country or other regions. It can also be followed to compare COVID-19 vulnerability and severity between its different variants. Moreover, the vulnerability and severity from different pandemics (e.g., COVID-19 versus H1N1) can be compared by using the methods followed in this study.

## Figures and Tables

**Table 1:** Multiple linear regression results for COVID-19 vulnerability.

	Coefficient	t-Value	*p*-Value
Constant	41.468	13.80	0.000
Coreness	4.579	0.279	0.781
Degree	−3175.511	−4.380	0.000
Closeness	−8667.510	−2.054	0.042
Betweenness	22.737	0.360	0.720
Eigenvector	−3.761	−0.296	0.768

Dependent variable: COVID-19 vulnerability.

**Table 2:** Multiple linear regression COVID-19 severity.

	Coefficient	*t*-Value	*p*-Value
Constant	−80.131	−0.97	0.334
Coreness	−0.098	0.000	1.000
Degree	1.46 × 10^5^	7.342	0.000
Closeness	−5.25× 10^4^	−0.452	0.652
Betweenness	−1423.047	−0.819	0.415
Eigenvector	−1379.437	−3.944	0.000

Dependent variable: COVID-19 severity.

**Table 3:** Comparison of R^2^ values between multiple linear regression and random forest regression.

	Multiple Linear Regression	Random Forest Regression
COVID-19 vulnerability	23.30%	82.44%
COVID-19 severity	35.80%	91.51%

## Data Availability

This study obtained research data from two publicly available sources: NSW Government’s COVID-19 data and statistics and online map services (Google Maps and OpenStreetMap).

## Supplementary Materials

The followings are available online at https://www.mdpi.com/article/10.3390/ijerph19042039/s1. Figure S1: A network consists of five edges and six nodes. Figure S2: The corresponding core–periphery structure of the network in Figure S1. The core nodes are e and f (shaded ones), and the peripheral nodes are a, b, c, and d. Table S1: Sociodemographic information of 137 postal areas [30]. Data were extracted at the postcode level.
