# Supplementary material for: Suburban Road Networks to Explore COVID-19 Vulnerability and Severity
Source: Int J Environ Res Public Health. 2022 Feb 11;19(4):2039. doi: 10.3390/ijerph19042039 (PMC8872200; doi:10.3390/ijerph19042039)
Supplement: Supplementary file 1 [file ijerph-19-02039-s001.zip › ijerph-1512452-supplementary.pdf]

Supplementary Materials

Table S1. Socio-demographic information of 137 postal areas [30]. Data were extracted at the postcode level.

Data Item	Minimum	Maximum	Average
Population	225	105,451	22,185.25
Median age	24	48	35.66
Gender (male)	44.70	66.20	49.68
Population per household	2.00	3.70	2.90
Median weekly income	785	2693	1727.88
Education (% of University graduate)	2.90	60.00	19.49
% of population born in Australia	23.20	83.70	54.79
Unemployment rate (%)	3.30	20.80	6.55
Tenure information (% of the population rented)	7.90	86.30	34.69

S1. Centrality Measures

Degree Centrality

$$C_D(u) = \frac{d_i}{(N-1)} \tag{1}$$

Where d_i is the degree of node u, and N is the number of nodes in the network [31].

Closeness Centrality

$$C_c(u) = \frac{N-1}{\sum_{y \in N} d(u,v)} \tag{2}$$

Where, N is the set of nodes in the network and $d(u,v)$ is the shortest path length between u and v [31].

Betweenness Centrality

$$C_b(u) = \sum_{s \neq v \neq t} \frac{\sigma_{st}(u)}{\sigma_{st}} \tag{3}$$

Where $\sigma_{st}(u)$ is the number of shortest paths between s and t that contain u, and σ_{st} is the shortest paths between s and t other than u [31].

Eigenvector Centrality

$$x_u = \frac{1}{\lambda} \sum_{v \in M(u)} x_v \tag{4}$$

Where v $\in M(u)$ is the sum of u such that u and v are connected, and λ is a constant [31].

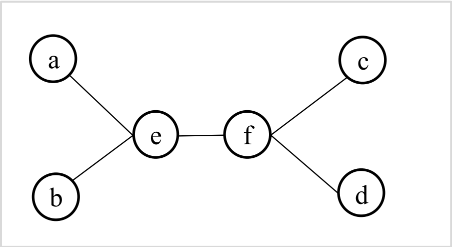

Figure S1. A network consists of five edges and six nodes.

Using formula 1, the degree values for different nodes of Figure S1 are:

Degree(a) = Degree(b) = Degree(c) = Degree(d) = $\frac{1}{6-1} = 0.200$

Degree(e) = Degree(f) = $\frac{3}{6-1} = 0.600$

Using formula 2, the closeness values for different nodes of Figure S1 are:

Closeness(a) = Closeness(b) = Closeness(c) = Closeness(d) = $\frac{6-1}{1+2+2+3+3} = 0.455$

Closeness(e) = Closeness(f) = $\frac{6-1}{1+1+1+2+2} = 0.714$

Using formula 3, the betweenness values for different nodes of Figure S1 are:

Betweenness(a) = Betweenness(b) = Betweenness(c) = Betweenness(d) = 0

[Since, these nodes do not fall in the shortest paths of any other pair of nodes]

Betweenness(e) = Betweenness(f) = $\frac{4+3+0+0}{4+3+2+1} = 0.700$

[For the betweenness centrality of node e: there are 10 shortest paths between other pairs of nodes of the network. Out of these 10 shortest paths, 7 of them contain node e. They are: aeb, aef, aefc, aefd, bef, befc and befd]

Using formula 4, the eigenvector values for different nodes of Figure S1 can be calculated by following multiple iterations:

Step 1: Calculate non-normalised degree values of different nodes (I, 3, 1, 1, 3 and 1)

Step 2: Calculate a normalised constant, C

$$c = \sqrt{1^2 + 3^2 + 1^2 + 1^2 + 3^2 + 1^2} = \sqrt{22} = 4.69$$

Step 3: Update degree values from Step 1 by dividing each of them with 4.69 to get a modified list of degree values:

$$\frac{1}{4.69}, \frac{3}{4.69}, \frac{1}{4.69}, \frac{1}{4.69}, \frac{3}{4.69}, \frac{1}{4.69}$$

Step 4: Repeat steps 2-3 until the value for C will converse (multiple iterations)

By following the above steps, we found that:

Eigenvector(a) = Eigenvector(b) = Eigenvector(c) = Eigenvector(d) = 0.408

Eigenvector(e) = Eigenvector(f) = 0.816

S2. Core-periphery Analysis

In a network, core nodes are those that have more connections with the remaining network nodes. On the other side, peripheral nodes are sparsely connections within network [31]. To get the core-periphery classification of nodes, we first need to remove nodes with a degree value of 0 (k = 0). Then, nodes with a degree value of 1 (k = 1) need to

be removed and so on. For k = 1, the corresponding core-periphery structure of Figure S1 is presented in Figure S2 (core nodes are shaded).

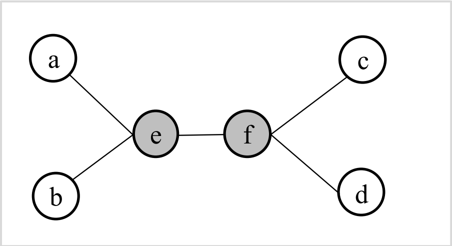

Figure S2. The corresponding core-periphery structure of the network in Figure S1. The core nodes are e and f (shaded ones), and the peripheral nodes are a, b, c and d.
